# Telomerase Reverse Transcriptase-Promoter Mutation in Young Patients with Bladder Tumors

**DOI:** 10.3390/cimb46040178

**Published:** 2024-03-23

**Authors:** Sonia Pérez González, Victoria Heredia-Soto, Manuel Girón de Francisco, Elia Pérez-Fernández, Rubén Casans-Francés, Marta Mendiola Sabio, Pilar González-Peramato

**Affiliations:** 1 Department of Urology, Infanta Leonor University Hospital, 28031 Madrid, Spain; 2 Translational Oncology Research Laboratory, Hospital La Paz Institute for Health Research (IdiPAZ), 28046 Madrid, Spain; 3 Center for Biomedical Research in the Cancer Network (CIBERONC), Instituto de Salud Carlos III, 28029 Madrid, Spain; 4 Department of Urology, La Paz University Hospital, 28046 Madrid, Spain; 5 Research Unit, Hospital Universitario Fundación Alcorcón, 28922 Madrid, Spain; 6 Department of Anesthesia and Pain Medicine, Infanta Elena University Hospital, 28342 Madrid, Spain; 7 Molecular Pathology and Therapeutic Targets Group, Hospital La Paz Institute for Health Research (IdiPAZ), 28046 Madrid, Spain; 8 Department of Pathology, La Paz University Hospital, 28046 Madrid, Spain; 9 Faculty of Medicine, Universidad Autónoma de Madrid, 28029 Madrid, Spain; 10 Cellular Engineering Laboratory, Hospital La Paz Institute for Health Research (IdiPAZ), 28046 Madrid, Spain

**Keywords:** *TERT*-promoter mutation, young patients, bladder cancer

## Abstract

The *TERT* (Telomerase Reverse Transcriptase) gene promoter mutation is one of the most prevalent mutations in urothelial bladder tumors and this mutation is related to bladder tumor progression. Our purpose was to evaluate the presence of this mutation in a population of patients who were first diagnosed at age ≤ 40 years and to examine its relationship with tumor characteristics and progression. A molecular study was performed to detect the two most prevalent mutations in the *TERT* promoter (C228T and C250T). The study included 29 patients, with a mean follow-up of 152 months. There were no statistically significant differences in the clinical or tumor characteristics according to the presence or absence of the mutation. Although the mutation group showed poorer recurrence-free survival (RFS), there was no statistically significant difference and there was no difference in progression-free survival by group (*p* > 0.05). The *pTERT* mutations in bladder tumor cells occurred less frequently in younger patients than in older patients, a finding that could indicate different mechanisms of carcinogenesis. The trend towards lower RFS in patients with mutated *pTERT* needs to be confirmed by further studies, given the small number of patients included in these studies due to the low incidence of bladder tumors in this age group.

## 1. Introduction

Telomerase is a ribonucleoprotein reverse transcriptase that performs telomeric DNA replication from an RNA template integrated into itself, stabilizing its length by preventing its shortening at each cell division [1,2]. The *TERT* gene encodes the catalytic subunit of telomerase responsible for its reverse transcriptase activity and is encoded on chromosome 5 [3,4]. The *TERT* gene promoter (*pTERT*) is a 260-base-pair (bp) region lacking a TATA box and containing binding motifs for various transcription factors, such as *p21*, *p53*, *E2F*, *AP1*, and *c-Myc*, whose binding to the promoter activates, to a greater or lesser extent, the transcription of the gene and thus the formation of its product, the catalytic subunit of telomerase. These transcription factors are involved in processes such as cell proliferation and growth, DNA synthesis, and apoptosis [5,6]. Based on a study by Chiba et al. published in 2017, it has been proposed that the mechanism by which the *pTERT* mutation has an impact on the increase in cell proliferation would have two phases: an initial phase in which telomere shortening would be slowed down, and another phase in which, when the telomere is sufficiently shortened, genomic instability would be generated that would induce the positive regulation of telomerase to maintain cell proliferation [7].

The most frequently detected tumor mutations in *pTERT* result in a C > T transition at 124 bp and 146 bp above the ATG transcription start site and are known as C228T and C250T mutations, respectively, according to their genomic coordinates (nucleotide 1,295,228 [C228T] and 1,295,250 [C250T]). These mutations result in an increase in *TERT* RNA expression and telomerase activity, which leads to the cells losing their limited proliferation capacity, becoming immortal and undergoing malignant cellular transformation [6,8,9,10,11,12,13,14,15].

Mutations in *pTERT* are frequently detected in various tumors, such as melanomas, gliomas, hepatocarcinomas, and papillary thyroid carcinomas [15,16]. In the bladder, *pTERT* mutations are prevalent in urothelial carcinomas, with a varying rate between 60% and 80% depending on the series [8,10,11,17,18,19,20,21,22]. For Pietzak et al., the *pTERT* mutation was the most frequent mutation in non-muscle-invasive bladder tumors (present in 73% of cases), followed by *FGFR3* (49%), *KDM6A* (38%), *PI3KCA* (26%), *STAG2* (23%), *ARID1A* (21%), and *TP53* mutations (21%) [23]. 

Mutations of *pTERT* are frequent in bladder tumors in patients aged 60–70 years and these mutations seem to have prognostic implications. For this reason, the study of *pTERT* mutations is one of the most promising DNA markers in bladder cancer [24]. In young patients, however, they are anecdotal, and the number of patients included has been low due to the low incidence of bladder tumors in patients younger than 40 years, which varies between 1% and 2.4% depending on the published series [25].

Our aim was to estimate the prevalence of *TERT* core promoter mutations in young patients with bladder tumor (first diagnosis at 40 years of age or younger) and to assess the differences in clinical tumor characteristics and disease progression.

## 2. Materials and Methods

### 2.1. Study Design and Sample Selection

We conducted a retrospective study by reviewing the medical records of patients up to 40 years of age diagnosed with bladder carcinoma and treated at our hospital between 1990 and 2007. The project was approved by the hospital’s Medical Research Ethics Committee. All the patients underwent transurethral resection of the bladder tumor. We selected cases with enough histological material for molecular study, with the material fixed by immersion in buffered formalin for 24–48 h. All samples included in the study were selected from hematoxylin and eosin-stained sections that were reevaluated by an expert pathologist who confirmed the diagnosis with histological stage and grade assignment according to the 2017 TNM classification (Union for International Cancer Control) [26]. We selected suitable tumor areas for study, with at least 50% tumor cells. 

### 2.2. Mutation Analysis

For *pTERT*-specific mutation analysis studies, the first step was DNA extraction from paraffin-embedded tissue. The material was extracted with the Qiamp DNA FFPE Tissue Kit (Qiagen, Hilden, Germany), and fluorometric quantification was performed with Qubit (ThermoFisher Scientific, Waltham, MA, USA). To identify the most prevalent mutations (C228T and C250T), specific polymerase chain reactions were performed with primers F: 5’CCCACGTGCGCAGCAGGAC and R: 5’CTCCCAGTGGATTCGCGGGC from Merck (Darmstadt, Germany) and subsequent Sanger sequencing for mutation identification. Chromatograms were analyzed with FinchTV software version 1.5.0 (Geospiza Inc., Denver, CO, USA). 

### 2.3. Evaluations

We assessed the demographics, personal and family history, exposure to risk factors, symptom presentation, tumor characteristics, treatment, progression, and the presence and type of *pTERT* mutations in tumor tissue cells.

### 2.4. Statistical Analysis

The statistical analysis was performed with SPSS 26.0. The quantitative data distribution was described using the mean and standard deviation (SD) or median and interquartile range (IQR). The qualitative data distribution was described using counts and relative frequencies. A univariate exploratory analysis was performed to compare clinical and pathological characteristics by group, with Fisher’s exact test in the case of qualitative data and the non-parametric Mann–Whitney U test in the case of quantitative data. Recurrence-free survival (RFS) and progression-free survival (PFS) were estimated using the Kaplan–Meier method, and a log-rank test was performed to compare survivor functions by group. Recurrence was defined as the diagnosis of a new tumor during follow-up. Progression was defined as the diagnosis of a new tumor with increased grade or stage during follow-up or the development of metastatic disease. To assess whether there was an age point at which *pTERT* mutations were more frequent, the cutoff point that maximized sensitivity and specificity for the receiver operating characteristic (ROC) curve was calculated.

## 3. Results 

### 3.1. Epidemiological and Clinical Parameters

A total of 43 patients were registered; however, 6 patients were excluded due to the lack of sufficient histological material for molecular study. Of the 37 included patients, a valid *pTERT* mutation molecular study was achieved in 29. Nine patients had a *pTERT* mutation; 100% of the mutations detected were C228T.

The mean age of the overall study population was 34.1 (±4.7 SD) years. The group of patients with mutated *pTERT* had a higher mean age of 35.8 years (±5.2 SD); however, no statistically significant differences were detected (*p* > 0.05). Most of the patients had no relevant personal history (Table 1). Smoking was the only risk factor detected, with no statistically significant differences depending on the presence or absence of mutated *pTERT*. The most prevalent clinical manifestation at diagnosis was macroscopic hematuria. Cytology was negative in most of the patients with non-mutated *pTERT*, showing a statistical difference with respect to those with mutated *pTERT*. Ultrasonography was the most frequently employed complementary diagnostic technique in both patient groups. The most frequent tumor location was on the left lateral wall of the bladder in the non-mutated *pTERT* group and on the posterior wall of the bladder in the mutated *pTERT* group, the latter being the only location with a statistically significant difference between the two groups. Four patients had tumors with more than one localization.

Regarding the tumors’ infiltrative behavior, 79.3% of the patients had a non-muscle-infiltrating tumor at diagnosis: 85% (n = 17) and 66.7% (n = 6) of the non-mutated and mutated *pTERT* groups, respectively. Six patients had a muscle-infiltrating tumor, equally distributed in both groups. Radical cystectomy with urinary diversion was performed in five (83.3%) of these cases, and palliative treatment was performed in the remaining patient because of their significantly deteriorated general condition. Low-grade tumors were present in 76.9% of the patients at diagnosis (Table 1).

### 3.2. Disease Evolution 

**Figure 1:**
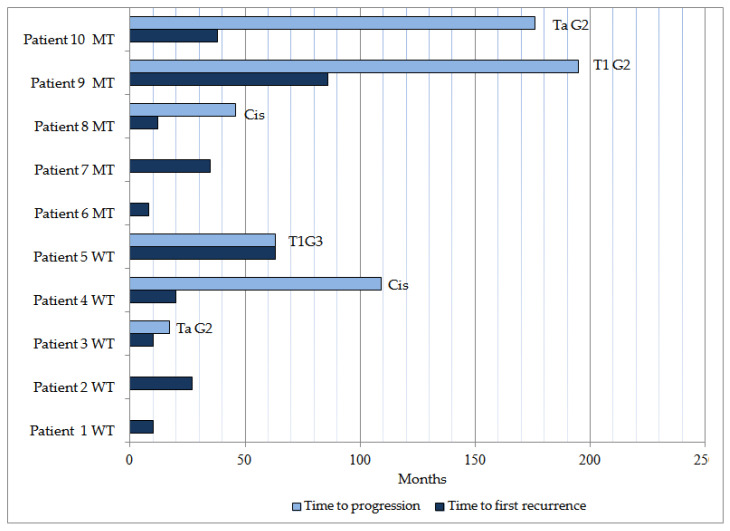
Patients with tumor recurrence. In cases where there was progression, the month of recurrence in which the progression was identified is indicated. Each case shows whether the tumor diagnosed was mutated (MT) or non-mutated/wild type (WT) *pTERT*.

The median follow-up was 152 (IQR 66–219) months, and two patients had TaG1 stage/grade loss after surgery, one of them with *pTERT* mutation. In the patients with non-muscle-infiltrating tumor at diagnosis, a total of ten recurrences were detected, five in each group of patients under study. All recurrences occurred in patients with a TaG1 tumor at diagnosis. Recurrences were unique in two patients, both with non-mutated *pTERT*. Tumor progression was detected in three patients in each group. Progression in both groups throughout the entire follow-up was according to grade in four patients and to grade and stage in two patients (Figure 1). None of the progressions were due to a muscle-infiltrating tumor. In the patient group with muscle-infiltrating tumors at diagnosis, three patients developed multiple metastases during the disease follow-up and were undergoing chemotherapy at the last known review (two patients in the *pTERT* non-mutated group and one in the *pTERT* mutated group). One patient with poor general condition at diagnosis and *pTERT* mutation was referred to the hospital’s palliative care service and died a few weeks after diagnosis. The remaining two patients with muscle-infiltrating tumors at diagnosis were free of disease at the last known review.

### 3.3. Survival

**Figure 2:**
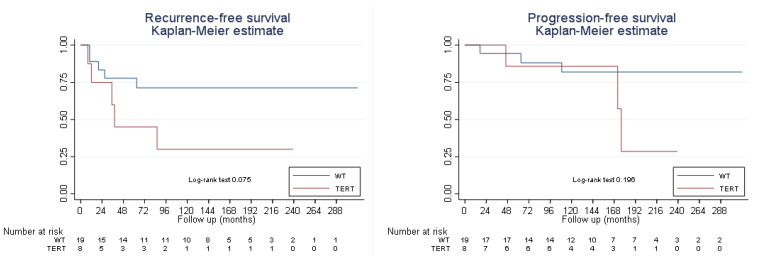
Recurrence-free survival and progression-free survival by *TERT* core promoter mutation state. Abbreviations: WT, wild-type *pTERT* or *pTERT* non-mutated group; TERT, *pTERT* mutated group.

RFS at 24 and 60 months was 83.3% (95% CI 56.8–94.3%) and 77.8% (95% CI 51.1–91.5%), respectively, in the non-mutated *pTERT* group. In the mutated *pTERT* group, the RFS was 75.0% (95% CI 31.5–93.1%) and 45.0% (95% CI 10.8–75.1%), respectively. Although the mutated group showed poorer RFS, there were no statistically significant differences (*p* > 0.05) (Figure 2), and there were no differences in PFS by group (*p* > 0.05). In the non-mutated *pTERT* group, PFS at 60 months was 94.4% (95% CI 66.6–99.2%); in the *pTERT* mutated group, PFS was 85.7% (95% CI 33.4–97.9%). 

**Figure 3:**
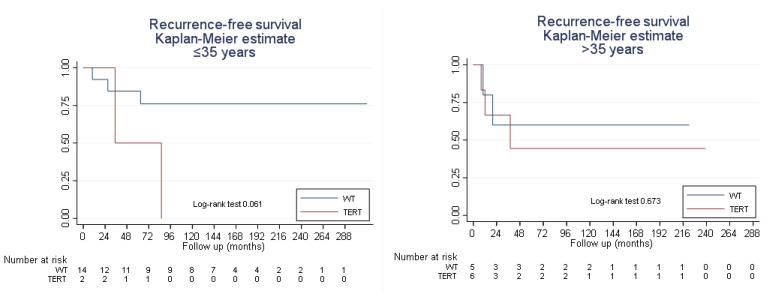
Recurrence-free survival related to the *TERT* core promoter mutation state in patients ≤35 years and >35 years of age. Abbreviations: WT, wild-type *pTERT* or *pTERT* non-mutated group; TERT, *pTERT* mutated group.

The study group was stratified into 17 patients aged ≤ 35 years and 12 patients between 36 and 40 years, according to the cutoff point that maximized sensitivity and specificity for the calculated ROC curves, identifying that the rate of *pTERT* mutation was significantly lower in those patients aged ≤ 35 years compared with those in the group aged > 35 years: 2 (11.8%) vs. 7 (58.3%) patients, respectively (*p* < 0.05). However, although the results appeared to show a greater impact of the mutation on recurrence in patients aged ≤ 35 years than in those aged > 35 years, no significant differences in outcome between the two groups were demonstrated (Figure 3).

## 4. Discussion 

In recent decades, there has been an increased number of studies on genetic alterations in various types of tumors, with the aim of detecting those alterations with significance in terms of tumor characteristics and/or behavior in order to personalize treatments or follow-ups. There have been numerous studies of the *pTERT* mutation in various tumors to detect its involvement in carcinogenesis, prognosis, or the prediction of response to various treatments. 

Most reported studies on the general population with bladder tumors detected no statistically significant differences in the percentage of patients with tumors with mutated *pTERT* according to the patients’ age. Roggisth et al. found no differences in their study of 75 patients divided by <75 and ≥75 years of age (49–97 years) [27]. Similar findings were reported by Siraj et al. in their study of patients aged ≤ 60 years or >60 years in which, although a higher number of mutations was found in the older age group, it was not significant [20]. Jahnson et al., in a recent study of patients with stage T1 bladder tumors, in a cohort of patients divided according to age ≤ 74 years or >74 years, showed no significant differences in the percentage of mutated *pTERT* [28]. In contrast, Wu et al., in their study of bladder tumors in patients aged < 50 years or ≥50 years, reported a higher percentage of tumors with mutated *pTERT* in the latter group (37.5% vs. 60%) [22].

Giedl et al. supported these findings in their study of 144 patients aged ≤ 45 (range 11–45) years and 125 patients from a consecutive cohort of patients with a mean age of 70.7 years (range 29–94), reporting a *pTERT* mutation rate of 57.6% vs. 84.8%, respectively, with a statistically significant difference. Within the age group of ≤45 years, the patients were stratified into two groups (<40 years and 40–45 years), with a statistically significant (*p* < 0.05) lower mutation rate in the patient group younger than 40 years: 46.3% (31/67) vs. 67.5% (52/77) [29]. In our study, the rate of *pTERT* mutations was 31% (nine patients), slightly lower than that reported in the previously cited study. Stratifying the study group into patients aged ≤ 35 years (17 patients) and 36–40 years (12 patients), the mutation percentage was significantly lower in those ≤35 years, at 11.8% vs. 58.3% (*p* < 0.05), which would be in line with the groups with a lower *pTERT* mutation rate at younger ages.

This lower frequency of genetic alterations in younger patients compared to the general population may be due to the fact that exposure to many urothelial carcinogens from occupational exposure or the direct inhalation of tobacco smoke is restricted to adults and that the time interval between exposure and subsequent carcinogenesis leading to tumor development is several years. Another possible explanation for this lower frequency of mutations in *pTERT* could be due to a lower or different exposure to microorganisms and their metabolites that may alter the functional state of tissues mediated by chronic inflammation and the immune system and thus contribute to the development of tumors in this group of patients [24,30]. Various studies performed on bladder tumors in a population with a mean age of approximately 65–75 years detected no relationship between sex and rate of *pTERT* mutations [8,20,27,28]. Our series also found no association between sex and *pTERT* mutation (*p* = 0.675) (Table 1). According to Wu et al., there is a tendency towards a higher rate of mutated *pTERT* in men than in women [22]. No study has been identified reporting differences in *pTERT* mutation by gender in young patients with bladder tumor.

The question of whether the frequency of *pTERT* mutations is correlated with tumor grade and/or stage is controversial, given that the mutations are present from early stages of tumor progression and in all tumor grades, and have been detected in high-grade and low-grade non-infiltrating urothelial bladder tumors, papillary urothelial neoplasia of low malignant potential (PUNLMP), carcinoma in situ (CIS), and at an advanced stage in muscle-invasive urothelial bladder tumors [14,18,27,31]. For a number of authors, these *pTERT* mutations were not associated with tumor grade or stage. A meta-analysis of 7259 cases detected a mutation rate for various tumor stages of 46% (44/95) in PUNLMP, 72% (1369/1895) in Ta–T1, and 70% (1404/1893) in muscle-invasive tumors (≥T2), with no significant differences between the various stages (Ta–T4). In relation to grade, the detected mutation rate was 70% in both high-grade and low-grade non-muscle-infiltrating tumors, with no significant differences [10]. However, other studies have shown an association between the mutation and tumor stage, such as that performed by Wu et al., who found that the frequency of the mutation is higher in muscle-invasive tumors [22]. 

Other studies, also conducted in patient groups of multiple ages, have reported an inverse association, i.e., tumors with mutated *pTERT* had lower stage and/or grade. For example, Siraj et al. detected a significant inverse relationship between *pTERT* mutation and bladder tumor metastasis. The authors also reported a higher mutation rate in low-stage tumors; however, this was not statistically significant. Other authors have reported no differences between the various grades [20]. In a study of 527 patients, 373 with Ta and 154 with T1 or higher, Eich et al. detected a *pTERT* mutation rate of 77% in low-grade Ta and 65% in high-grade Ta and CIS, with a statistically significant difference (*p* < 0.05). The authors also found that stage T1 tumors had a higher rate of mutations than T2 tumors (72% vs. 63%); however, this difference was not statistically significant [32]. Kindle et al. also reported a higher rate of mutated *pTERT* in low-grade Ta tumors, compared with high-grade and CIS tumors (86%, 68%, and 65%, respectively); however, this was not statistically significant [18].

Although mutations were detected in muscle-invasive and non-muscle-invasive tumors in our study, there was no statistically significant association between the stage and rate of *pTERT* mutation in tumor cells (*p* > 0.05). Similarly, *pTERT* mutations were detected in low-grade and high-grade tumors, but with no statistically significant differences (*p* > 0.05) (Table 1).

A number of studies have reported that *pTERT* mutations are related to survival and recurrences of urothelial bladder carcinomas, with survival being more favorable in patients with tumors with low mutational burden, and tumor recurrences more frequent in tumors with *pTERT* mutations. In a meta-analysis published in 2021 with 1382 patients included from various studies performed in tissue or urine cells, Wan et al. reported a *pTERT* mutation rate of 62.5% and showed a significantly higher recurrence rate in cases with mutated *pTERT*; however, the authors found no association between mutation and overall survival (OS) [21]. Although other authors have been unable to demonstrate a larger number of recurrences in low-grade bladder tumors with mutated *pTERT*, they have been able to demonstrate a larger number of recurrences in PUNLMP [33]. In our case, the recurrence rate showed a higher tendency in cases of tumors with a *pTERT* mutation, although with no statistically significant differences (Table 1). However, we cannot discard the possibility that the absence of significance could be due to the relatively low number of samples. 

The findings on the relationship between *pTERT* mutation and disease recurrence and progression could allow for the optimization of patient follow-up by detecting these mutations using non-invasive or minimally invasive methods such as blood or urine samples. It has been shown that there is a very high correlation (>90%) between *pTERT* mutations observed in urine-derived urothelial cells and those present in bladder tumors in the same patients. Patients in whom the mutation was not detected in urine cells mostly had low-grade tumors [34]. Regarding the sensitivity and specificity of *pTERT* mutation detection in urinary cells, Descotes et al. performed a study detecting an overall sensitivity of 80.5% and a specificity of 89.8%, not significantly affected by inflammation or infection [35]. Similar results were obtained by Dahmcke et al. in a study of 475 patients, with a sensitivity and specificity of 81.8% and 83.5%, respectively [36]. In cases where *pTERT* remained positive after initial surgery, this was associated with residual carcinoma in situ. Likewise, *pTERT* mutation determination in urine was a good predictor of recurrence in non-invasive tumors, such that *pTERT*-positive status was associated with residual carcinoma in situ. Furthermore, the determination of *pTERT* mutation in urine was a good predictor of recurrence in non-invasive tumors, such that positive *pTERT*-mutated urine status after initial surgery increased the risk of recurrence 5.34-fold, and contributed to the detection of recurrences even in patients with normal cystoscopy [35].

In relation to OS, Roggisch et al. observed no significant difference in survival in patients with mutated *pTERT*, although the tendency was for OS and specific cancer to worsen in those patients with mutated *pTERT* [27]. Wu et al. detected a significantly shorter OS time in patients with mutated *pTERT*, while Isharwal et al. observed a shorter disease-free survival in patients with mutated *pTERT* [13,22]. Lastly, other studies have concluded that there was no significant relationship between the mutation or non-mutation of *pTERT* with either OS, cancer-specific survival, or recurrence [8,18,28,32].

Regarding the relationship between *pTERT* mutation and tumor progression, there are discordant results; however, most studies have found no association between the two variables [18,28,31]. The findings in our series are concordant and showed no association between progression and *pTERT* mutation (Table 1).

As in the other tumor types in which mutated *pTERT* has been detected, the most frequently identified *pTERT* mutation in urothelial carcinomas was C228T (80–90%), followed by the C250T mutation (3–4%) [8,13,14,16]. In our case, all the detected mutations were of the C228T type, which is in line with previously reported findings.

Our study has limitations, such as the discrete number of patients. Other limitations are due to the complexity involved in performing molecular studies on tissue samples preserved in paraffin for many years, and to the lower rate of bladder cancer patients in this age group (≤40 years). However, the study of TERT could also be important in this population. While previously mentioned studies are contradictory about the prognostic value of *TERT* mutation determination, very recent meta-analyses have concluded that the *TERT* may be a valuable marker for individual prognostic value, as *TERT* mutation increases the risk of death and decreases the survival time of urothelial carcinoma patients [37]; in addition the presence of TERT-promoter mutations could even negatively affect the outcome of urothelial carcinoma patients treated with chemotherapy or immunotherapy [38].

## 5. Conclusions

The mutation of *pTERT* in bladder tumor cells is prevalent in the general population; however, the rate of mutations in young patients was clearly lower in our series and in those of the few reported studies in this age group, which could indicate that the *pTERT* mutation and its repercussions are not as influential in the development of carcinomas in young patients. Based on our data, tumors with mutated *pTERT* show a tendency to present a higher recurrence rate and earlier recurrences than non-mutated tumors in this patient group, which is why their follow-up should be intensified. These results should be confirmed by further studies that demonstrate findings along these lines, given the small number of study patients due to the low incidence of bladder tumors in this age group.

## Figures and Tables

**Table 1:** Differences in clinical and pathological characteristics by *pTERT* mutation.

Clinical and Pathological Characteristics	Total	WT *pTERT*	Mutated *pTERT*	*p*-Value
No. of patients	29	20 (69%)	9 (31%)	
Sex				
Male	21 (72.4%)	15 (75%)	6 (66.7%)	0.675
Female	8 (27.6%)	5 (25%)	3 (33.3%)	
Age, years; mean (SD)	34.1 (4.7)	33.3 (4.2)	35.8 (5.2)	0.107
Personal Background				
Smoker	19 (65.5%)	14 (70%)	5 (55.6%)	0.675
HBP	1 (3.4%)	1 (5%)	0	1
DL	1 (3.4%)	1 (5%)	0	1
DM	1 (3.4%)	1 (5%)	0	1
Other	10 (34.5%)	6 (30%)	4 (44.4%)	0.68
ASA				
1	22 (75.9%)	17 (85%)	5 (55.6%)	0.212
2	5 (17.2%)	2 (10%)	3 (33.3%)	
3	2 (6.9%)	1 (5%)	1 (11.1%)	
Cytology				
Positive	7 (24.1%)	3 (15%)	4 (44.4%)	0.005
Suspicious	2 (6.9%)	0	2 (22.2%)	
Negative	20 (69%)	17 (85%)	3 (33.3%)	
Clinical diagnosis				
Macroscopic hematuria	26 (89.7%)	18 (90%)	8 (88.9%)	1
Urinary syndrome	3 (10.3%)	2 (10%)	1 (11.1%)	1
Location				
Trigone	4 (13.8%)	2 (10%)	2 (22.2%)	0.568
Posterior wall	5 (17.2%)	1 (5%)	4 (44.4%)	0.022
Right wall	6 (20.7%)	5 (25%)	1 (11.1%)	0.633
Left wall	14 (48.3%)	12 (60%)	2 (22.2%)	0.109
Anterior wall	2 (6.9%)	0	2 (22.2%)	0.089
Neck—posterior urethra	3 (10.3%)	2 (10%)	1 (11.1%)	1
Stage at diagnosis (T)				
Ta	22 (75.9%)	16 (80%)	6 (66.7%)	0.744
T1	1 (3.4%)	1 (5%)	0	
T2	4 (13.8%)	2 (10%)	2 (22.2%)	
T3	2 (6.9%)	1 (5%)	1 (11.1%)	
Grade at diagnosis (G)				
PUNLMP	3 (10.3%)	3 (15%)	0	0.478
Carcinoma G1	20 (69%)	14 (70%)	6 (66.7%)	
Carcinoma G3	6 (20.7%)	3 (15%)	3 (33.3%)	
Tumor size, cm; median (IQR)	2 (1–3)	2 (1–2)	2 (1–4)	0.358
Recurrence	10 (34.5%)	5 (25%)	5 (55.6%)	0.12
Progression	6 (20.7%)	3 (15%)	3 (33.3%)	0.339

Abbreviations: HBP, high blood pressure; DL, dyslipidemia; DM, diabetes mellitus; ASA, American Society of Anesthesiologists physical status; PUNLMP, papillary urothelial neoplasm of low malignant potential; WT *pTERT*, wild-type *pTERT* or *pTERT* non-mutated group; SD, standard deviation; IQR, interquartile range.

## Data Availability

Data are available from the research team upon request.
